# Atmospheric Light Estimation Using Polarization Degree Gradient for Image Dehazing

**DOI:** 10.3390/s24103137

**Published:** 2024-05-15

**Authors:** Shuai Liu, Hang Li, Jinyu Zhao, Junchi Liu, Youqiang Zhu, Zhenduo Zhang

**Affiliations:** 1 Changchun Institute of Optics, Fine Mechanics and Physics, Chinese Academy of Sciences, Dong Nanhu Road 3888, Changchun 130033, China; shuailiu@ciomp.ac.cn (S.L.); hangli@ciomp.ac.cn (H.L.); zhuyouqiang@ciomp.ac.cn (Y.Z.); 2 Navigation College, Dalian Maritime University, Linghai Road 1, Dalian 116026, China; zhangzhenduo@dlmu.edu.cn

**Keywords:** atmospheric light estimation, near-zero closure interval for polarization, polarization degree gradient, dehazing

## Abstract

A number of image dehazing techniques depend on the estimation of atmospheric light intensity. The majority of dehazing algorithms do not incorporate a physical model to estimate atmospheric light, leading to reduced accuracy and significantly impacting the effectiveness of dehazing. This article presents a novel approach for estimating atmospheric light using the polarization state and polarization degree gradient of the sky. We utilize this approach to enhance the outcomes of image dehazing by applying it to pre-existing dehazing algorithms. Our study and development of a real-time dehazing system has shown that the approach we propose has a clear advantage over previous methods for estimating ambient light. After incorporating the proposed approach into existing defogging methods, a significant improvement in the effectiveness of defogging was noted through the assessment of various criteria such as contrast, PSNR, and SSIM.

## 1. Introduction

The presence of fog leads to an increased concentration of scattering particles in the atmosphere, causing light to undergo attenuation and directional deviations when interacting with these particles. This phenomenon can impact the quality of visual system imaging, resulting in challenges such as reduced luminosity, lower contrast, and blurred distant objects in images captured under foggy conditions. Improving the quality of images affected by fog is of significant practical importance for imaging systems [1,2,3,4,5]. Current research on defogging methods primarily focuses on enhancing light transmission, often using approximate techniques to estimate atmospheric light values. The accurate estimation of atmospheric light values is crucial as it influences the projection function’s outcome, affecting image realism and potentially causing color distortion. The importance of precise atmospheric light value estimation in the defogging process is highlighted by the potential color distortion in resulting images [6,7,8,9,10].

Most of the methods for estimating atmospheric light have been developed in conjunction with algorithms for image dehazing. He et al. [11] utilized the dark channel prior method to calculate the transmission map and estimated atmospheric light by identifying the brightest point in the dark-channel image of the fogged scene. However, this approach fails to consider the impact of high-brightness pixels in the scene, leading to a significant bias in atmospheric light estimation for specific images. Kim [12] was the first to employ a quadtree hierarchical search method to estimate atmospheric light values, based on the observation of minimal pixel variance in hazy images. This method helps prevent misinterpretation of high grayscale value pixels as atmospheric light, which could result in excessive contrast enhancement [13,14]. Compared to He’s technique [11], Kim’s method substantially reduces errors in estimating atmospheric light caused by bright objects in the image, although it may not be suitable for images with dense fog. Another approach, as outlined in [15], utilizes clustering statistics to estimate atmospheric light values by analyzing clusters of light source points. Wang et al. [16] proposed a method to estimate atmospheric light by examining geometric relationships among haze lines in the three-dimensional RGB space using Plücker coordinates, providing a precise analytical solution for determining atmospheric light. However, this method requires extensive data sampling to ensure relatively accurate estimation results, leading to increased computational costs and potentially yielding locally optimal outcomes instead of globally optimal ones. Matteo’s method [17] relies on statistical analysis of common atmospheric light colors from natural image datasets to initiate a minimization process of a cost function. This algorithm adjusts atmospheric light parameters to minimize the cost function and determine the intensity of atmospheric light.

In recent years, there has been a growing number of dehazing methods proposed that leverage deep learning techniques. The initial deep learning model used for dehazing, known as DehazeNet [18], has been criticized for its imprecise estimation of atmospheric light values, necessitating further improvements to enhance the dehazing results. Yang and colleagues introduced a novel approach [19] that utilizes Convolutional Neural Networks (CNNs). A key feature of this method is the incorporation of a bilinear composition loss function, which explicitly accounts for the interactions among the transmission map, clear image, and atmospheric light. This design allows for the simultaneous error back-propagation to each sub-network, while maintaining the composition constraint to prevent overfitting in individual sub-networks. The proposed technique has shown remarkable performance in effectively processing heavily noisy and compressed haze images.

Studies have shown that natural environments display distinct variations in energy levels based on different polarization states, spectra, and coherence characteristics between the sky background and objects within the scene [20,21]. This research presents a new method for predicting atmospheric light intensity by utilizing gradient data obtained from polarization images of natural scenes. The main aim is to enhance the effectiveness of existing dehazing algorithms that rely on precise estimation of atmospheric light intensity.

## 2. Related Works

### 2.1. Image Degradation Model

In conditions of limited visibility, incident light reaching the sensor can be divided into two distinct components. The first component comprises direct light emanating from the object of focus and penetrating through the haze, while the second component is the airlight, which is the scattered light diffused by the haze particles [22,23,24,25]. As a result, the light intensity captured by a camera can be expressed mathematically as follows:

(1) I=D+A

where *A* is indicative of the airlight. The direct light, denoted by *D*, is the attenuated form of the object light at a specific distance *z*, with the object light serving as the desired signal for recovery. The parameter *A* demonstrates a rise as the distance increases, and when *A* is quantified from an infinite distance, it is frequently symbolized as $A_{\infty }$. Consequently, Equation (1) can be rephrased as follows:

(2) $$I=L\cdot t\left(z\right)+A_{\infty }\cdot \left(1-t\left(z\right)\right)$$

where t(z) represents the attenuation phenomenon occurring with distance, often known as the atmospheric transmittance. By substituting t(z) with D/L in Equations (1) and (2), the dehazed image *L* can be determined as follows:

(3) $$L=\frac{I-A}{1-A/A_{\infty }}$$

When both *A* and $A_{\infty }$ are obtained, it becomes feasible to generate a clear image that is devoid of haze interference.

### 2.2. The Stokes Representation of Linear Polarization

The proposed approach relies on calculating linear polarization within images. The Stokes vector [26] that characterizes the scene can be determined by employing polarization images of the scene taken with polarization orientations set at $0^{\circ }$, $45^{\circ }$, $90^{\circ }$, and $135^{\circ }$, labeled as I(0), I(45), I(90), and I(135). The representation of the scene’s Stokes vector can be articulated as follows:

(4) $$S=\left[\begin{array}{c} S_{0}\\ S_{1}\\ S_{2} \end{array}\right]=\left[\begin{array}{c} \frac{1}{2}\left(I(0)+I(45)+I(90)+I(135)\right)\\ I(0)-I(90)\\ I(45)-I(135) \end{array}\right]$$

In the Stokes vector framework, $S_{0}$ signifies the overall intensity of the scene, $S_{1}$ indicates the disparity in intensity between horizontally and vertically polarized light, and $S_{2}$ represents the difference in intensity between light polarized at angles of $45^{\circ }$ and $135^{\circ }$. Within polarization dehazing methodologies, the degree of polarization (DoP) serves as a physical parameter that reflects the proportion of polarized light intensity to the total light intensity within the scene. The polarized light derived from $S_{1}$ and $S_{2}$ can be expressed as follows:

(5) $$S_{p}=\sqrt{S_{1}^{2}+S_{2}^{2}}$$

The overall matrix representation of the linear polarization degree for the scene can be expressed as follows:

(6) $$DoP=\frac{S_{p}}{S_{0}}$$

### 2.3. Influence of $A_{\infty }$ in Dehazing Algorithm

As demonstrated in Equation (3), the influence of the global atmospheric light $A_{\infty }$ on the dehazing outcome is evident. To investigate the effect of atmospheric light intensity on the dehazing process, we methodically modified the estimations of atmospheric light for the DCP (Dark Channel Prior) [11], Fattal’s [23] dehazing algorithm, and our previously proposed dehazing method [27], by adjusting the values in both decreasing and increasing directions.

**Figure 1:**
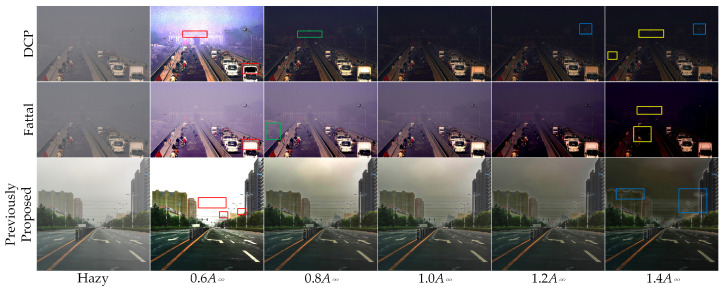
The dehazing results of the three dehazing methods at different estimated global atmospheric light values. (**top**): The impact of global atmospheric light intensity on original dark channel dehazing method. (**middle**): The impact of global atmospheric light intensity on Fattal’s dehazing. (**bottom**): The impact of global atmospheric light intensity on our previously proposed method.

The atmospheric light intensity values for the three algorithms were modified to 0.6, 0.8, 1.2, and 1.4 times their estimated values. Figure 1 depicts the dehazing results in response to changes in atmospheric light intensity. A decrease in atmospheric light intensity results in a general rise in luminosity. At 0.8 times the atmospheric light intensity, the three algorithms demonstrate improved contrast and brightness in comparison to utilizing the estimated atmospheric light intensity. This enhancement is particularly evident in the green rectangular areas within the image. Nevertheless, when the atmospheric light intensity diminishes to 0.6 times, the images tend to become overexposed. In the red rectangular areas of the image, the target appears submerged as a result of overexposure. Conversely, a rise in atmospheric light intensity leads to a general reduction in luminosity. When the atmospheric light intensity reaches 1.4 times its normal level, the brightness values in specific areas of the image decrease significantly, making it difficult to distinguish objects in the images. This phenomenon is particularly evident in the yellow rectangular regions. Elevating atmospheric light intensity in both DCP and our previously proposed method results in the emergence of prominent halos at the edges of objects. This effect is particularly noticeable in the blue rectangular regions of the image.

The precise determination of the global atmospheric light, referred to as $A_{\infty }$, is a critical factor in the efficacy of dehazing processes. A precise estimation of the global atmospheric light is imperative for achieving superior dehazing outcomes. Despite this importance, many dehazing algorithms rely on estimating the overall atmospheric light through an analysis of image intensity distribution. Typically, the estimation of atmospheric light intensity is based on the maximum image intensity value or the maximum value of a specific color channel. This approach lacks robust physical significance in certain images, leading to an overestimation of atmospheric light intensity. Consequently, this results in decreased brightness in the dehazed image and diminished contrast between the foreground and background.

## 3. Atmospheric Light Estimation Theory

### 3.1. Polarization Analysis of Natural Scenes

In natural environments, the brightness observed in the sky portion is primarily a result of atmospheric illumination. Therefore, accurately identifying and delineating the sky area can greatly enhance the precision of estimating atmospheric light intensity within dehazing algorithms.

In our previous research [27], an examination was conducted on the polarization properties observed in images of natural environments. Following a thorough analysis of polarization and statistical features using a substantial dataset of polarized images, encompassing more than 500 samples, the study has revealed the subsequent outcomes. Generally, the polarization level of atmospheric light appears to be lower in comparison to that of natural objects. The polarization degree of atmospheric light is almost negligible when contrasted with the polarization degree exhibited by natural objects. This observation holds true for images with both clear visibility and those affected by haze.

The polarized images utilized in our study were sourced from two distinct origins. A segment of the data was acquired from the PolarLITIS polarized image dataset (https://zenodo.org/records/5547760), accessed on 20 December 2023. Furthermore, we employed a proprietary polarization detector equipped with the Sony IMX250 (Tokyo, Japan) sensor chip to capture the remaining collection of images.

**Figure 2:**
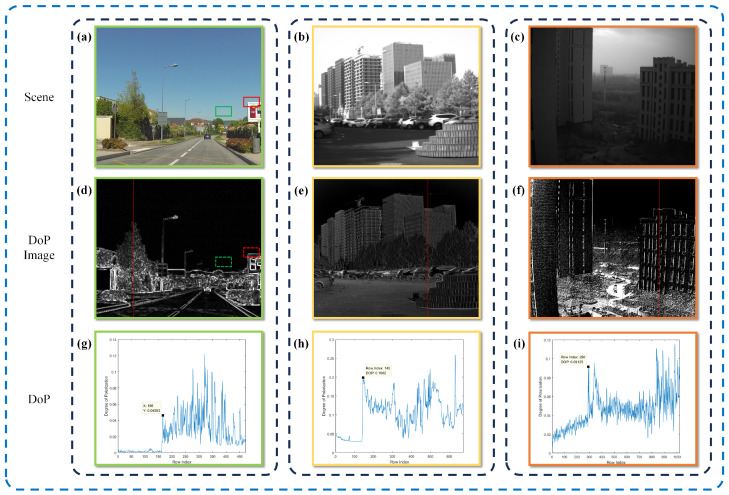
(**a**–**c**) The intensity image obtained from the synthesis of four polarized images. (**d**–**f**) The degree of polarization (DoP) image of (**a**–**c**). (**g**–**i**) DoP map of the columns marked with red lines in (**d**–**f**).

Figure 2 displays three images showcasing different sky regions. The first image (Figure 2a) is a color image sourced from the PolarLITIS dataset, while the subsequent grayscale images (Figure 2b,c) were captured by our detector. It is noteworthy that the polarimetric properties of the sky region exhibit a marked contrast with those of the overall scene, highlighting objects with pronounced polarization characteristics. In particular, the polarimetric degree of the sky region tends towards zero.

The green rectangular portion depicted in Figure 2a displays a greater luminance in comparison to the red rectangular portion. However, it is evident that the green rectangular area exhibits a lower level of polarization when contrasted with the red area, as illustrated in Figure 2d. Certain existing image dehazing methodologies face difficulties in precisely estimating the atmospheric light intensity. These methods tend to focus on the region with the highest intensity within the entire image, potentially resulting in the use of intensity values from an incorrect area as the estimated global atmospheric light intensity. This scenario may cause a darkening effect in the dehazed image, with noticeable halos possibly appearing at the edges of objects. It is essential to confine the atmospheric light intensity to the specific region, which in this case corresponds to the green rectangular area.

The polarization curves for the columns highlighted by the red lines in Figure 2d–f are presented in Figure 2g–i. A noticeable trend is observed as the transition occurs from the sky area to the object area, with a general increase in the degree of polarization. Analysis of the polarization curves reveals that the degree of polarization is predominantly close to zero in the sky region. A marked rise in polarization is evident at the interface between the sky and objects, indicating a distinct edge effect in the polarization degree image.

**Figure 3:**
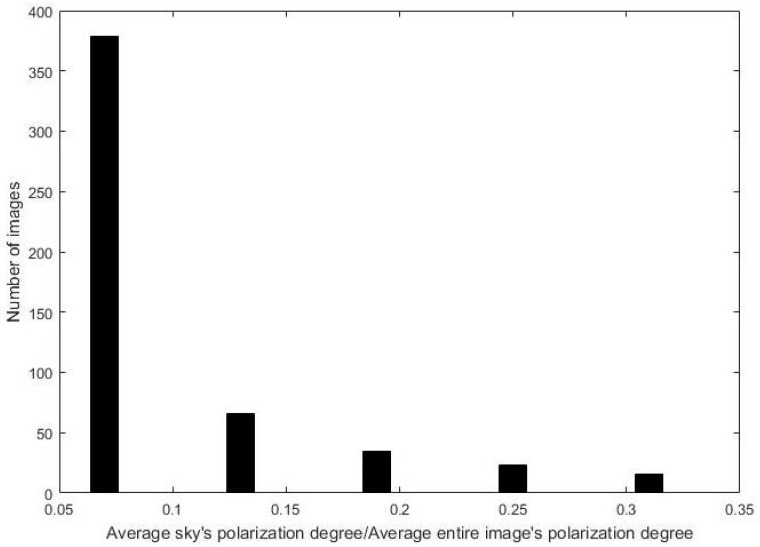
The statistics of the polarimetric degree in the sky region.

Figure 3 illustrates the distribution of the measurement ratio across 517 nature scene photographs. An examination of more than 350 images reveals that the ratio between the polarimetric degree of the sky area and the overall polarimetric degree of the image is typically below 10%. A minority of images exhibit ratios surpassing thirty percent, with each individual image maintaining a ratio below one. This finding suggests that the polarimetric intensity of the sky region constitutes a relatively minor component of the total polarimetric intensity of the image. Leveraging this notable distinction allows for the effective identification of sky regions in natural photographs. Subsequently, this approach is applied to estimate the atmospheric light *A* within the dehazing algorithm that relies on atmospheric light estimation. This refinement enhances the method’s adaptability and facilitates a more accurate determination of atmospheric light levels.

### 3.2. Sky Region Near-Zero Closure Interval for Polarization

According to the examination conducted in Section 3.1, the notion of the near-zero closure interval for polarization (PZCI) is introduced. The term is used to describe contiguous regions within natural scene images where the level of polarization is in close proximity to zero.

In the polarization degree image of natural scenes, the PZCI must demonstrate the following attributes:

(1) This range encompasses the pixel exhibiting the minimum intensity within the polarization degree image.
(2) This specific range within the polarization degree image forms a contiguous and enclosed area, encompassed by pixels exhibiting higher polarization degrees in the surrounding vicinity.

**Figure 4:**
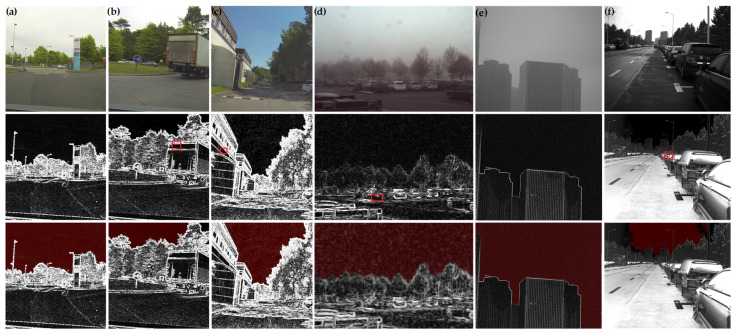
Polarization characteristics of natural scenes. (**a**–**d**) Images from dataset, PolarLITIS. (**e**–**f**) Images captured by our system. (**top**): Original images of natural scenes. (**middle**): Polarization degree images after exponential stretching. (**bottom**): Near-zero closure interval for polarization. **Red rectangles**: Near-zero polarization degree regions in non-sky area.

In Figure 4, the sections marked in red denote the PZCI, which serves to delineate the energy levels of the sky area. Subsequently, a methodology will be introduced in the subsequent section for ascertaining the PZCI, which will be utilized for estimating the overall atmospheric luminosity.

## 4. Polarization Degree Gradient Model for Estimation of $A_{\infty }$

In order to apply our methodology, it is essential to obtain energy images in four distinct polarization directions, labeled as $i_{0},i_{45},i_{90}$, and $i_{135}$, for an image of a natural scene. Subsequently, the polarimetric degrees for every pixel within the acquired image are determined through the utilization of Equations (4)–(6). This computational procedure yields a visual representation known as the Degree of Polarization (DoP) map.

### 4.1. Polarization Degree Image Preprocessing

In Section 3.1, an examination was carried out on the characteristics of the polarization degree as it approaches zero within the celestial domain of images portraying natural landscapes. Nevertheless, practical observations reveal the presence of pixels exhibiting low polarization values in specific sections of the object, as indicated by the red rectangular areas in the polarization degree illustration in Figure 4. This occurrence complicates the identification of the pixel with the minimum polarization degree. Consequently, it is imperative to preprocess the polarization degree image by removing the locally low-polarization regions within the object area.

The noise present in the polarization degree image is predominantly found in high-frequency signals, primarily concentrated within the object region, leading to the potential occurrence of locally extremely low polarization values. This could be identified as pseudo-edges. The application of a Gaussian filter convolution to the original polarization degree image results in a reduction of interference from individual pixels, thereby decreasing the probability of misidentifying them as the area with the lowest polarization degree. Concurrently, this procedure improves the precision of edge detection within PZCI.

(7) $$G\left(x,y\right)=\frac{1}{2\pi \sigma^{2}}\exp\left(-\frac{x^{2}+y^{2}}{2\sigma^{2}}\right)$$

(8) $$H_{ij}=\frac{1}{2\pi \sigma^{2}}\exp\left(-\frac{{\left(i-\left(k+1\right)\right)}^{2}+{\left(j-\left(k+1\right)\right)}^{2}}{2\sigma^{2}}\right);\;1\leq i,\;j\leq \left(2k+1\right)$$

The process of enhancing the image of the polarization degree involves applying a Gaussian kernel through convolution. The generating function for the Gaussian kernel is represented by Equation (7). The discrete version of Equation (7) is presented in Equation (8). In these equations, the variables *i* and *j* refer to distances in two directions from the center of the kernel, while 2k+1 indicates the size of the Gaussian kernel. The parameter σ denotes the standard deviation of the Gaussian filter. A higher value of σ results in a broader distribution of the convolution kernel. This broader distribution causes the image to appear more blurred and filtered, retaining fewer fine details but improving the ability to eliminate local extremes.

**Figure 5:**
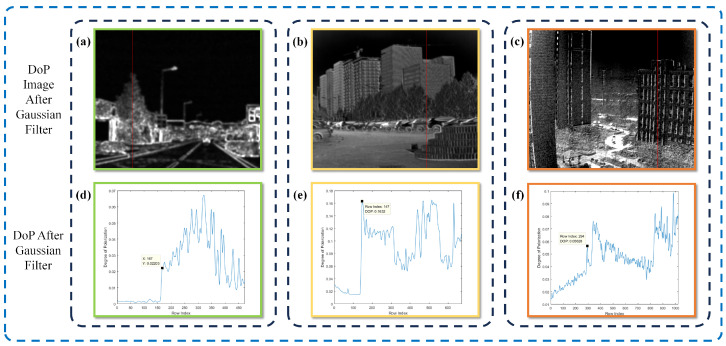
(**a**–**c**) Gaussian filter images of Figure 2d–f. (**d**–**f**) DoP map of the columns marked with red lines in (**a**–**c**).

Figure 5 demonstrates the results achieved through the utilization of Gaussian filtering with parameters σ=2 and k=8 on the polarization degree images depicted in Figure 2. Analysis of sections a-c reveals that the edge details within the scene are preserved post the application of Gaussian filtering to the polarization degree image. Simultaneously, the object area effectively eliminates the extremely low values. Graph the polarization degree curves for the column highlighted in red. In comparison to Figure 2g–i, the polarization degree displays a more uniform appearance, and the very low polarization values within the object area are raised, ensuring that the minimum polarization values in the image are concentrated in the sky region. Additionally, the enhancement of gradients at the boundary between the sky and the object becomes more pronounced.

### 4.2. Locating a Pixel within PZCI

According to the feature described in Section 3.2, pixels with lower polarization values are first detected within the polarization degree image. Following this, pixels belonging to the PZCI are identified within these locations, distinguished by having the lowest polarization values.

In our previous work [27], a technique was introduced for estimating atmospheric light through the utilization of polarization degree imagery. The approach involves identifying pixel locations characterized by the lowest polarization values within PZCI. The method involves detecting pixels that display the lowest 0.1% polarimetric degree in the polarization image and recording their respective coordinates. In practical application, the presence of detector noise may introduce inaccuracies in the calculation of sky polarimetric states, potentially leading to errors in polarimetric degree metrics, as well as in the automated identification of erroneous sky areas and the estimation of atmospheric light. To mitigate these challenges, specific adjustments were made to the algorithm. The process for determining the polarimetric degree is delineated as follows:

(9) $$\hat{p}\left(x,y\right)=\frac{\sum_{i\in \Omega_{xy}}p_{i}}{N_{xy}}$$

where $\hat{p}\left(x,y\right)$ refers to the adjusted polarimetric degree of the pixel at coordinates (x,y). The notation $\Omega_{xy}$ is used to denote the spatial region surrounding the pixel (x,y). In this context, p(x,y) represents the polarimetric degree of the corresponding pixel in the initial polarimetric degree image, and $N_{xy}$ indicates the number of pixels within the vicinity $\Omega_{xy}$.

The minimum response energy of the polarimetric detector imposes constraints that result in low pixel grayscale values when incident light is weak. This can lead to similar signal intensities across the four polarized angles, causing the calculated polarimetric degree to approach zero. As a result, non-sky regions may exhibit lower polarimetric degrees compared to sky regions. The presence of areas in the image with low signal pixels exceeding 0.1% of the total pixel count can introduce inaccuracies in estimating atmospheric light intensity. To address this issue, enhancements have been implemented in the technique for estimating atmospheric light. A threshold denoted as θ is introduced to identify pixels with polarimetric degrees below this threshold, which are then recorded and included in the process of determining the initial estimation of atmospheric light intensity using the pixel *S*.

(10) $$\bar{p}=\frac{\sum \hat{p}}{N}$$

(11) $$p_{t}=\theta \times \bar{p}$$

(12) $$C_{low}=\left\{\left(x,y\right)|{\hat{p}}_{x,y}\leq p_{t}\right\}$$

(13) $$S=\left(i_{c},i_{r}\right)=\left\{\left(x,y\right)|e_{x,y}=max\left(C_{low}\right)\right\}$$

In accordance with the information provided in Section 3.1, it is observed that the polarimetric degree of atmospheric light in images generally constitutes less than 10% of the average polarimetric degree across the entire image. To accommodate the algorithm’s tolerance, the parameter θ was set at a value of 0.15. Initially, the average polarimetric degree of the image denoted as $\bar{p}$ is computed. Subsequently, the calculation of $p_{t}$ is executed, followed by the documentation of the coordinates of all pixels with a polarimetric degree lower than $p_{t}$. The brightest pixel at these identified coordinates in the input image is then designated as *S*. Equations (10)–(13) are employed for the determination of the pixel *S*, which serves as the preliminary estimation of atmospheric light intensity at the coordinates $\left(i_{c},i_{r}\right)$. Here, the variable *N* represents the total pixel count in the image, while ${\hat{p}}_{x,y}$ denotes the polarimetric degree at the specific coordinate (x,y). Furthermore, $e_{x,y}$ signifies the maximum intensity value among the pixels in $C_{low}$. In accordance with the delineation of PZCI characteristics expounded in Section 3.2, pixel *S* is situated within the PZCI region.

### 4.3. Identifying PZCI and $A_{\infty }$

The exploration of the perimeter of PZCI commences at the initial position labeled as *S*. The perimeter is recognized as the location where a change in pixel intensities takes place. This examination entails the use of gradients in both the horizontal and vertical directions of the pixels to quantify the extent of variation in pixel intensities. A significant change in gradient values indicates the probability of a pixel being indicative of an edge pixel.

(14) $$G=\sqrt{G_{x}^{2}+G_{y}^{2}}$$

(15) $$\alpha =arctan(\frac{G_{y}}{G_{x}})$$

The symbol *G* represents the magnitude of the gradient, while α indicates the direction of the gradient, which is the angle measured counterclockwise relative to the horizontal axis. The symbols $G_{x}$ and $G_{y}$ represent the gradient magnitudes of the pixel along the horizontal and vertical axes, respectively. The Sobel operator is employed for the computation of gradients $G_{x}$ and $G_{y}$. It can be formally described as follows:

(16) $$K_{x}=\left[\begin{array}{ccc} -1 & 0 & 1\\ -2 & 0 & 2\\ -1 & 0 & 1 \end{array}\right]\;,\;K_{y}=\left[\begin{array}{ccc} 1 & 2 & 1\\ 0 & 0 & 0\\ -1 & -2 & -1 \end{array}\right]$$

In the context of edge detection, the Sobel operator $K_{x}$ is applied in the *x* direction, while the Sobel operator $K_{y}$ is applied in the *y* direction. A 3×3 window *W* is defined, and convolution operations are performed using $K_{x}$ and $K_{y}$ as kernels on each pixel within the polarimetric image.

(17) $$\begin{array}{c} G_{x}=K_{x}*W=\left[\begin{array}{ccc} -1 & 0 & 1\\ -2 & 0 & 2\\ -1 & 0 & 1 \end{array}\right]*\left[\begin{array}{ccc} p_{x-1,y-1} & p_{x,y-1} & p_{x+1,y-1}\\ p_{x-1,y} & p_{x,y} & p_{x+1,y}\\ p_{x-1,y+1} & p_{x,y+1} & p_{x+1,y+1} \end{array}\right]\\ =sum\left(\left[\begin{array}{ccc} -p_{x-1,y-1} & 0 & p_{x+1,y-1}\\ -2p_{x-1,y} & 0 & 2p_{x+1,y}\\ -p_{x-1,y+1} & 0 & p_{x+1,y+1} \end{array}\right]\right)\\ =-p_{x-1,y-1}+p_{x+1,y-1}-2p_{x-1,y}+2p_{x+1,y}-p_{x-1,y+1}+p_{x+1,y+1}\\ G_{y}=K_{y}*W=\left[\begin{array}{ccc} 1 & 2 & 1\\ 0 & 0 & 0\\ -1 & -2 & -1 \end{array}\right]*\left[\begin{array}{ccc} p_{x-1,y-1} & p_{x,y-1} & p_{x+1,y-1}\\ p_{x-1,y} & p_{x,y} & p_{x+1,y}\\ p_{x-1,y+1} & p_{x,y+1} & p_{x+1,y+1} \end{array}\right]\\ =sum\left(\left[\begin{array}{ccc} p_{x-1,y-1} & 2p_{x,y-1} & p_{x+1,y-1}\\ 0 & 0 & 0\\ -p_{x-1,y+1} & -2p_{x,y+1} & -p_{x+1,y+1} \end{array}\right]\right)\\ =p_{x-1,y-1}+2p_{x,y-1}+p_{x+1,y-1}-p_{x-1,y+1}-2p_{x,y+1}-p_{x+1,y+1} \end{array}$$

The process for determining the horizontal and vertical polarimetric gradient values at a specific pixel location (x,y) is described in Equation (17). The function sum is employed to compute the total sum of elements within the convolution-produced matrix. Following the convolution operation on the complete polarimetric degree image, the polarimetric gradient magnitudes and orientations for all pixel coordinates are obtained through Equations (14) and (15). This gradient data is then utilized to define the Polarimetric PZCI.

In the Gaussian filtering procedure discussed in Section 4.1, there is a possibility of amplification of edges. To address this issue, the non-maximum suppression method [28] is employed to eliminate points that do not represent edges, with the objective of maintaining the edge width as close to one pixel as possible. According to the requirement, if a pixel lies on an edge, its gradient magnitude should exceed the gradient magnitudes of its neighboring pixels in the gradient direction. Pixels failing to meet this condition are not identified as edge pixels.

**Figure 6:**
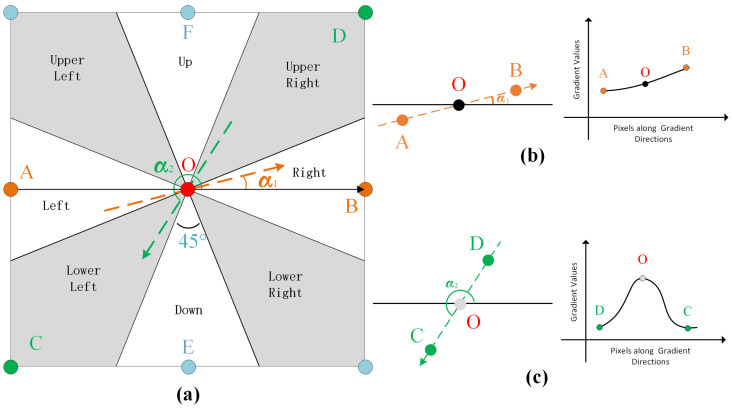
(**a**) Neighboring pixels along the gradient direction. (**b**) Retained pixel after non-maximum suppression. (**c**) Eliminated pixel after non-maximum suppression.

Figure 6 illustrates the concept of non-maximum suppression technique. The region surrounding pixel O is divided into eight sectors, namely top, top-left, left, bottom-left, bottom, bottom-right, right, and top-right, each dividing the circular angle centered at O equally. By determining the sectors in which the gradient direction vector of a pixel lies, it becomes possible to identify neighboring pixels along the gradient direction. For instance, when the gradient direction of pixel O is denoted as $\alpha_{1}$ and shown by the orange dashed line, its neighboring pixels along the gradient are A and B. Similarly, if the gradient direction of pixel O is $\alpha_{2}$, represented by the green dashed line, its neighboring pixels along the gradient are C and D. In another scenario depicted in Figure 6b, pixels C and D are identified as adjacent along the gradient direction of pixel O. Since the gradient magnitude of pixel O is not the highest among the three pixels, pixel O is classified as a non-edge pixel and therefore should be excluded. Conversely, in Figure 6c, pixels A and B are situated along the gradient direction of pixel O. As the gradient value of pixel O is the highest among the three pixels, pixel O is retained as a potential boundary pixel.

Following the processing described above, the pixels within the image that may indicate boundaries were preserved and put into set $C_{edge}$. However, $C_{edge}$ includes pixels that depict both object boundaries and edge artifacts caused by camera noise, as well as inaccuracies in polarimetric calculations. It is crucial to remove these pixels to isolate only those that accurately outline the boundaries of the sky area.

In Section 3.1, an examination of the polarization properties of natural images indicates that the level of polarization in the sky area is notably lower compared to the overall polarization level of the image. Additionally, in accordance with characteristic (2) of PZCI outlined in Section 3.2, a distinct contrast in the polarization level of pixels is observed between the sky area and its boundary. Leveraging this finding, a technique is introduced that combines pixel polarization degree and gradient data to detect the PZCI region. Algorithm 1 is a pseudo-code for determining the PZCI region.
**Algorithm 1** Determination of PZCI01.   initialize set $C_{pzci}$ to empty02.   initialize set $C_{bd}$ to empty03.   **procedure** PZCI_Search(*Dop Map*, *S*):04.         let *Q* be an empty queue05.         *Q*.enqueue(*S*) // explore from *S*06.         **while** *Q* is not empty **do** // when *Q* is empty, PZCI is determined07.         *V* = *Q*.dequeue()08.         **if** *V* is an internal pixel of PZCI **then** // based on Equation (18)09.               put *V* into set $C_{pzci}$10.         **else if** *V* is a boundary pixel of PZCI **then** // based on Equation (19)11.               put *V* into set $C_{bd}$12.               **continue** // stop exploring the neighboring pixels of *V*13.         **for** each neighboring pixel *W* of *V* **do**14.               **if** *W* is not in set $C_{pzci}$ nor set $C_{bd}$ **then**15.                     *Q*.enqueue(*W*)16.         **return** $C_{pzci}$

Before applying the algorithm described above, it is essential to assign a polarization degree of 1.0 to the pixels located at the edges of the DoP Map and include the corresponding pixels from the gradient image in the set $C_{edge}$. This procedure ensures the uniform treatment of these pixels during the algorithm’s assessment of whether they constitute PZCI edges. The evaluation in the DoP map begins at the pixel $S(i_{c},i_{r})$. Subsequent to this, the adjacent pixels are inspected to determine their involvement in the boundary of the sky region, following a breadth-first search sequence. For instance, as illustrated in Figure 6a, the pixel denoted as *O* is examined. If a pixel is not part of the sky region’s boundary, it is classified as an internal pixel of PZCI and added to the pixel set $C_{pzci}$. Further analysis is then conducted to confirm if the remaining unclassified pixels *A*, *E*, *B*, and *F* are boundary pixels. If a pixel is recognized as being on the boundary of the sky region, it is identified as an edge pixel of PZCI and assigned to the pixel set $C_{bd}$.

(18) $$C_{pzci}\subseteq \left\{\left(x,y\right)|{\hat{p}}_{x,y}<\bar{p}\left(x,y\right)\right\}\cup \bar{C_{edge}}$$

(19) $$C_{bd}\subseteq \left\{\left(x,y\right)|{\hat{p}}_{x,y}\geq \bar{p}\right\}\cap C_{edge}$$

The eighth step of the algorithmic sequence involves assessing pixels to determine their inclusion in the set $C_{pzci}$ by leveraging the principle of nearly zero polarization degree in the atmospheric light region and employing the maximum gradient suppression technique. Pixels with polarization degrees lower than the average polarization degree of the image and not part of the set $C_{edge}$ are identified as internal components of the PZCI region. The set $C_{pzci}$, derived from the aforementioned algorithm, encompasses all pixels within the PZCI region, while $C_{bd}$ consists of the boundary pixels of the PZCI region. Subsequently, the pixels in $C_{pzci}$ are arranged in a descending order based on their brightness in the dark channel. The top 0.1% of pixels are then chosen to form the set $C_{at}$.

**Figure 7:**
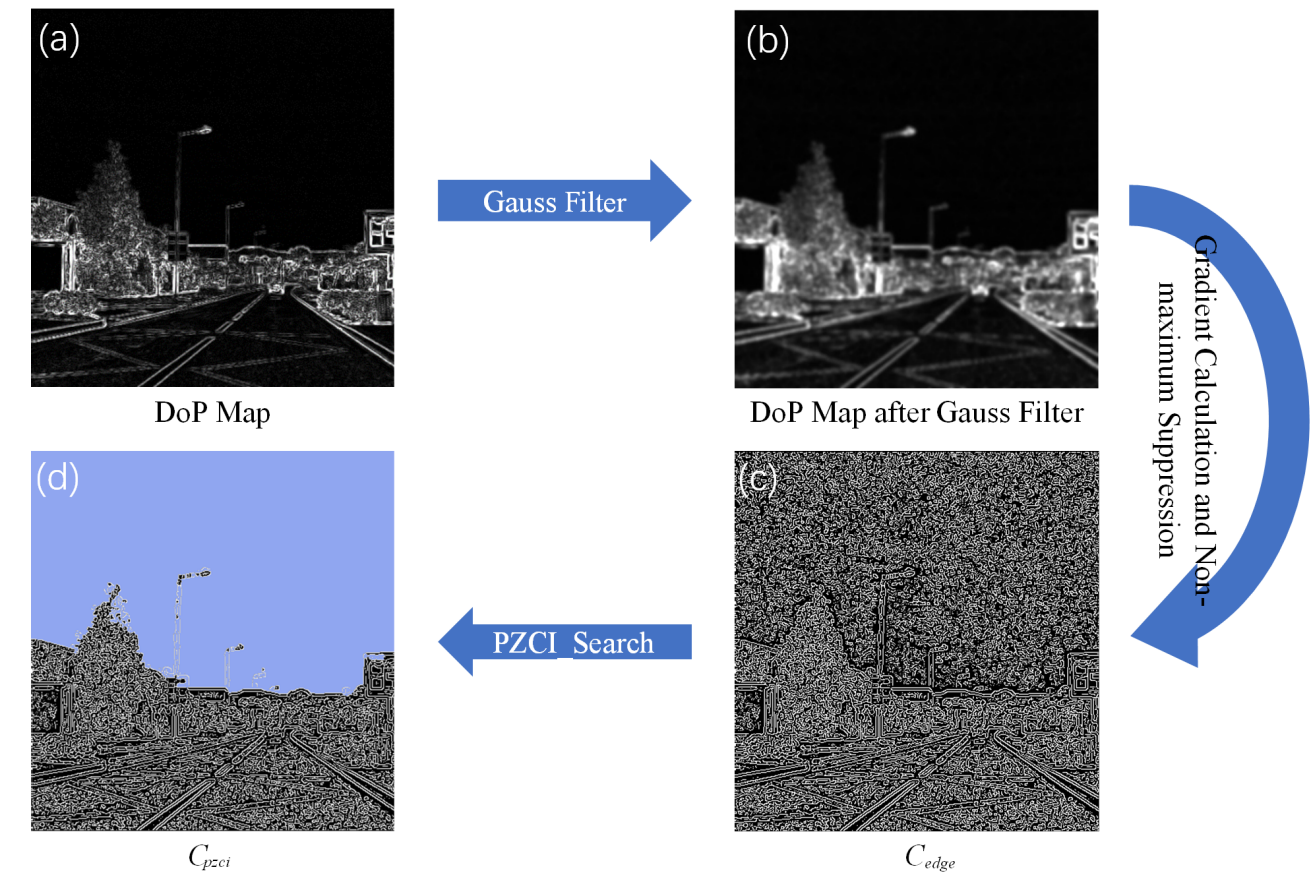
An execution example of PDGA. (**a**): DoP Map. (**b**): DoP Map after Gauss Filter. (**c**): Set $C_{edge}$ after gradient calculation and non-maximum suppression. (**d**): Set $C_{pzci}$.

The illustration in Figure 7 showcases the execution of the PDGA algorithm. After applying maximum gradient value suppression in Figure 7c, a significant number of pixels representing PZCI boundaries are retained in the $C_{edge}$ set. Subsequently, upon executing the PZCI_Search algorithm, $C_{pzci}$ retains all pixels situated within the PZCI regions, as indicated by the blue area in Figure 7d.

(20) $$A_{\infty }=\left[A^{r}A^{g}A^{b}\right]=\frac{\sum_{j\in C_{at}}\left[I_{j}^{r}I_{j}^{g}I_{j}^{b}\right]}{N_{C_{at}}}$$

The equation referenced as (20) is employed for the calculation of the atmospheric light denoted as $A_{\infty }$, where the mean pixel intensity within the designated set $C_{at}$ is utilized as an approximation of the total atmospheric light intensity. In this context, $I_{j}^{r},I_{j}^{g},I_{j}^{b}$ represent the three component values of pixel *j* in the RGB color space, and $N_{C_{at}}$ denotes the cardinality of the set $C_{at}$.

At this point, the PDGA has defined specific regions representing the global atmospheric light intensity and has provided an estimated value for the global atmospheric light intensity denoted as $A_{\infty }$. In the following section, we will apply $A_{\infty }$ in various traditional image dehazing algorithms that rely on atmospheric light intensity to demonstrate the advantages of the proposed algorithm.

## 5. Results and Discussion

In this segment, we introduce our experimental setup and perform deblurring trials on real-world scenes using the proposed method for estimating atmospheric light. To evaluate the effectiveness of our methodology, we compare it with several commonly used deblurring techniques, considering its advantages in enhancing image contrast, preserving fidelity, and improving signal intensity.

### 5.1. Experimental System

**Figure 8:**
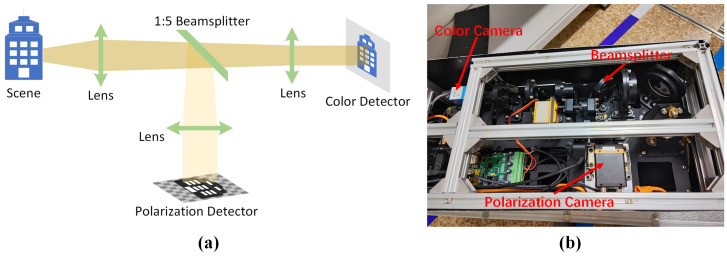
(**a**) Optical design of the system. (**b**) Internal structure of the equipment.

In order to achieve live polarization dehazing, our team of engineers has designed and implemented a haze elimination system that utilizes polarization gradient atmospheric light estimation. This system includes a telescope with a 150 mm aperture. Figure 8 depicts the optical layout and physical object of the system.

The system comprises two detectors: a black-and-white visible light polarization detector and a visual visible light imaging detector. The polarization detector utilizes a Sony-manufactured CMOS sensor, specifically the IMX250 model, which offers enhanced detection capability and quantum efficiency compared to the imaging detector. To ensure optimal performance in our optical setup, a beamsplitter is utilized to divide the incoming light between the polarization and imaging detectors, maintaining a 1:5 intensity ratio. This configuration ensures that the polarization detector does not become saturated, enabling the imaging detector to receive a greater amount of incident light energy.

**Figure 9:**
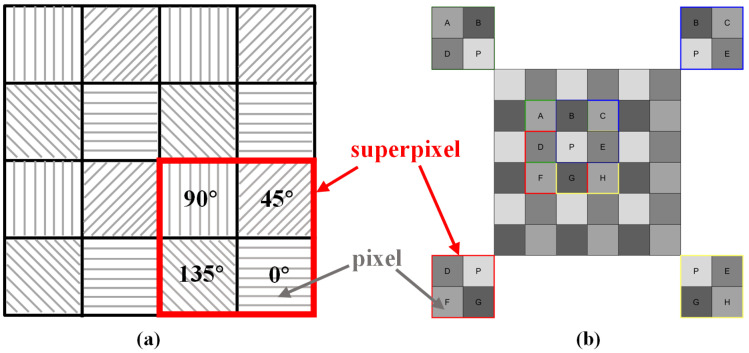
(**a**) Distribution of the polarization detector’s pixels. (**b**) Calculating the polarization degree of pixels.

In order to assess the level of polarization in an image, it is imperative to possess four intensity images corresponding to the four polarization orientations: 0°, 45°, 90°, and 135°. These four images necessitate precise alignment at the pixel level. The polarization sensor was designed utilizing the Sony IMX250 sensor, recognized for its capability to sense polarization at the pixel level. Each pixel on the sensor is equipped with a micro-polarizer. The sensor boasts a resolution of 2048×2048. Each group of pixels in a 2×2 configuration contains polarization information for all four directions. The arrangement of pixels in the sensor, depicted in Figure 9a, eliminates the need for employing an image alignment algorithm to achieve pixel-level alignment of the four images. Within the IMX250 sensor, each group forming a 2×2 array of four pixels, referred to as a superpixel, captures energy from all four polarization angles. Therefore, to maintain the original resolution as much as possible, calculations for the polarization state are conducted for each superpixel. Consequently, the resulting image representing the degree of polarization has a resolution of 2047×2047. As shown in Figure 9b, a pixel labeled as P in the original polarization image can be utilized in the polarization state computation to serve as an input for calculating four superpixels. Thus, the energy of pixel P contributes to these four superpixels.

### 5.2. Experimental Environment

The primary data used in the following experimental results is derived from two separate sources. A portion of the data comes from the experimental setup described in Section 5.1. The remaining data is sourced from the PolarLITIS polarization image dataset.

The experimental configuration consists of two main components. One component pertains to the image capture system, as detailed in Section 5.1, while the other component deals with the data processing system. The processing system operates on a computer equipped with an Intel(R) Core(TM) i7-9700 CPU (Santa Clara, CA, USA) running at 3.00 GHz, a NVIDIA GeForce RTX 3060 graphics card (Santa Clara, CA, USA), and 32 GB of RAM. The algorithm is developed using the Python (Version 3.6) programming language.

### 5.3. Objective Evaluation

The contrast, Peak Signal-to-Noise Ratio (PSNR), and Structural Similarity Index Measure (SSIM) [29] were utilized to appraise the quality of images after dehazing. Contrast was employed to evaluate the differentiation between foreground and background in images before and after dehazing, serving as a direct measure of the dehazing technique’s effectiveness. PSNR can be used to assess image quality and the efficacy of dehazing at the pixel level, with a higher numerical value indicating better image quality. SSIM quantifies image similarity by analyzing brightness, contrast, and structure, with a higher numerical value indicating better preservation of structural information. The contrast calculation formula is represented as Formula (21), the PSNR calculation formula as Formula (22), and the SSIM calculation formula as Formula (23).

(21) $$C=\sum_{\delta }\delta {\left(i,j\right)}^{2}P_{\delta }\left(i,j\right)$$

where δ(i,j) is the grayscale difference between adjacent pixels, and $P_{\delta }\left(i,j\right)$ is the probability distribution of pixels with a grayscale difference of δ between adjacent pixels.

(22) $$PSNR=10\cdot \log_{10}\frac{Max^{2}}{MSE}$$

where Max is the highest brightness value of the image pixels, and MSE is the mean square error between the dehazed image and the corresponding hazy image.

(23) $$SSIM\left(J,I\right)=l{\left(I,J\right)}^{\alpha }+c{\left(I,J\right)}^{\beta }+s{\left(I,J\right)}^{\gamma }$$

In Formula (23), the input hazy image is denoted as *I* and the dehazed image is represented by *J*. The weighting coefficients α, β, and γ are uniformly set to a value of 1. The functions *l*, *c*, and *s* correspond to luminance similarity, contrast similarity, and structure similarity, respectively. These functions are defined as follows:

(24) $$\begin{array}{cc} l(I,J) & =\frac{2\mu_{I}\mu_{J}+\epsilon_{1}}{\mu_{I}^{2}+\mu_{J}^{2}+\epsilon_{1}}, \end{array}$$

(25) $$\begin{array}{cc} c(I,J) & =\frac{2\sigma_{IJ}+\epsilon_{2}}{\sigma_{I}^{2}+\sigma_{J}^{2}+\epsilon_{2}} \end{array}$$

(26) $$\begin{array}{cc} s(I,J) & =\frac{\sigma_{IJ}+\epsilon_{3}}{\sigma_{I}\sigma_{J}+\epsilon_{3}} \end{array}$$

In the context of statistical analysis, the mean values of variables *I* and *J* are denoted by $\mu_{I}$ and $\mu_{J}$, respectively, while the standard deviations of these variables are represented by $\sigma_{I}$ and $\sigma_{J}$, respectively. The covariance between variables *I* and *J* is denoted by $\sigma_{IJ}$. To address potential calculation errors resulting from a denominator being zero, the constants $\epsilon_{1}$, $\epsilon_{2}$, and $\epsilon_{3}$ are employed.

### 5.4. Experimental Results

**Figure 10:**
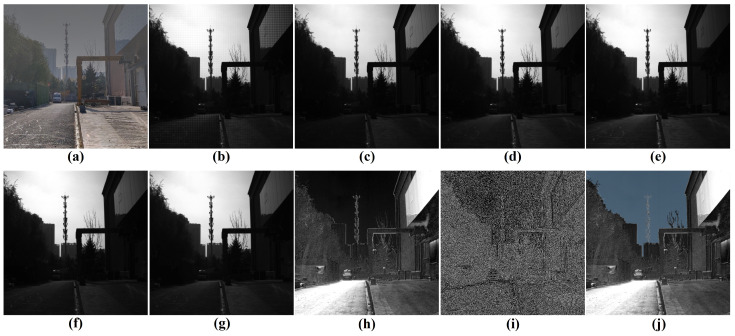
(**a**) Visual detector image. (**b**) Polarizatin detector image. (**c**–**f**) Intensity images of four polarization angles obtained after polarization image decomposition. (**g**) Intensity image composed from (**c**,**f**) using Equation (4). (**h**) DoP map. (**i**) $C_{edge}$ image. (**j**) PZCI determined by the proposed approach.

Figure 10 displays intensity images corresponding to four distinct polarization angles and a degree of polarization image captured by the polarization detector. The analysis of Figure 10h reveals a notable decrease in polarization levels within the sky region. Particularly, the region outlined by the green rectangle exhibits the lowest degree of polarization, with pixel point S displaying the minimum polarization degree. Figure 10i is the $C_{edge}$ image derived from the polarization gradient calculation and non-maximum suppression applied to the polarization degree image. Furthermore, Figure 10j demonstrates the effective identification of the atmospheric light region through the proposed method. This approach utilizes the intensity value of this region in the original image as an estimate for the atmospheric light.

**Figure 11:**
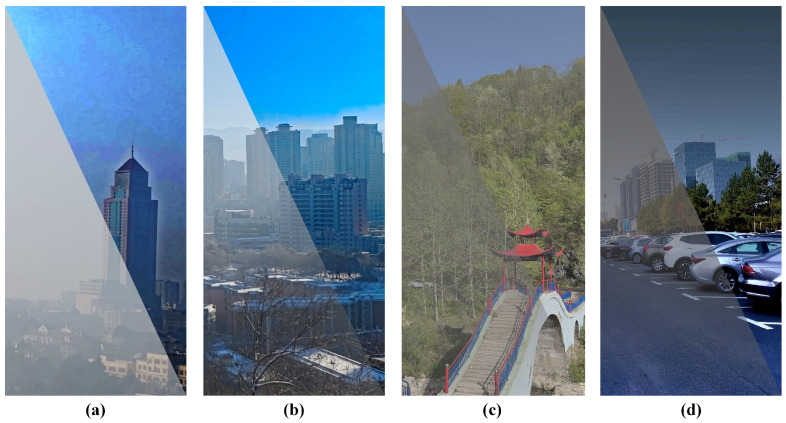
The performance of proposed approach in existing dehazing algorithm. (**a**) DCP with PDGA. (**b**) Fattal’s.with PDGA. (**c**) GPLF with PDGA. (**d**) Our previously proposed algorithm with PDGA.

Figure 11 demonstrates the results of applying the PDGA method to four established dehazing algorithms: Dark Channel Prior [11], Fattal’s Single Image Dehazing [23], GPLF [30], and a previously introduced polarimetric dehazing technique [27]. The lower-left portions of the images show the original hazy images, while the upper-right sections display the results after dehazing. It is apparent that the integration of the proposed method in dehazing algorithms, which traditionally depend on estimating atmospheric light intensity, effectively preserves the dehazing capability of the algorithm while ensuring the stability of the original method remains intact. Subsequently, the proposed method will be applied to the aforementioned four dehazing algorithms, and the outcomes will be evaluated in comparison to the original algorithms.

#### 5.4.1. DCP with PDGA

Given that the method we previously introduced is a modification targeted at the atmospheric light estimation component of the original DCP, the outcomes achieved by implementing the proposed method on the original DCP and the previously propoesd method are identical.

**Figure 12:**
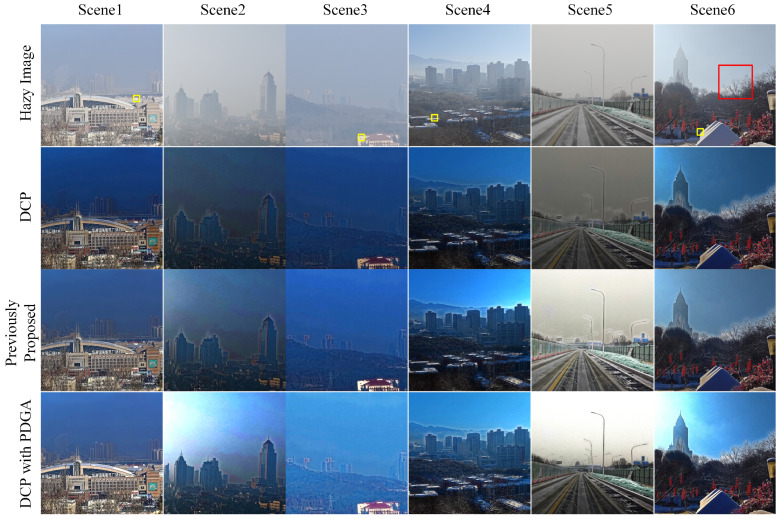
The performance of proposed approach in DCP.

Figure 12 illustrates the effects of the traditional Dark Channel Prior (DCP) method, a previously proposed technique, and the DCP enhanced with Polarization-Dark Channel Prior Guided Atmospheric Light Estimation (PDGA). The conventional DCP technique estimates the atmospheric light by identifying the brightest area in the dark channel. In Scenes 1, 3, 4, and 6 of Figure 12, the original DCP algorithm designates the yellow rectangular regions as the atmospheric light estimation areas. However, the selection of these regions by the original DCP method appears to lack consistency, as it is heavily influenced by the brightness of elements within the scene. This often results in a general darkening of the dehazed image, accentuating block artifacts along object boundaries.

**Figure 13:**
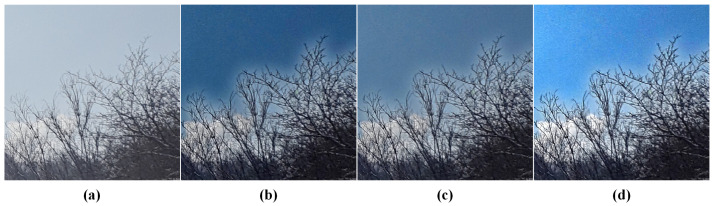
The zoomed-in views of the region of interest marked with red rectangle in Scene 6. (**a**) Hazy image. (**b**) Original DCP. (**c**) Previously proposed. (**d**) DCP with proposed approach.

Figure 13 provides detailed views of the magnified red rectangular section within Scene 6 of Figure 12. The presence of block artifacts is noticeable in Figure 13a when dehazing sky backgrounds with low contrast, resulting in unsatisfactory dehazing outcomes. These artifacts persist despite adjustments to parameters. A previously proposed method for determining atmospheric light intensity involves utilizing the intensity value of the pixel with the lowest degree of polarization in the polarization image. However, this method is vulnerable to noise in the polarization image, and the luminance of a single pixel is insufficient for accurately representing atmospheric light intensity. Although this method avoids estimating atmospheric light from scene elements, blocky artifacts continue to appear at object edges. The approach presented in this study addresses these issues by segmenting the sky area and calculating its average brightness to estimate atmospheric light. This approach effectively reduces the tendency of DCP to overestimate atmospheric light values in images containing sky regions and helps mitigate errors in atmospheric light estimation caused by noise in the polarization image. Consequently, this method enhances overall image brightness and reduces block artifacts along object edges, as demonstrated in Figure 13d.

Table 1 presents an objective evaluation of the haze removal method based on the dark channel prior (DCP). It is evident that integrating the proposed atmospheric light estimation method into the original DCP framework produces positive outcomes in contrast, PSNR, SSIM, and other evaluation metrics when applied to different natural images. A notable issue arises from the overestimation of atmospheric light intensity in the original DCP, resulting in a dark dehazing effect. This results in minimal improvement in contrast, as the brightness levels of the foreground and background become similar. Notably, the contrast enhancement observed in Scene 4 is primarily attributed to its noticeable edge block effect, where the sky area surrounding objects exhibits excessively high brightness. The overestimation of atmospheric light intensity by the original DCP has a significant impact on the Peak PSNR, leading to a darker overall dehazed image. The variation in brightness between the dehazed and hazy images results in a higher MSE, which in turn lowers the PSNR and indicates lower image quality. Although the method proposed earlier effectively avoids the incorrect selection of atmospheric light regions by the original DCP, the random selection of atmospheric light caused by polarized camera noise does not significantly improve the PSNR performance. While there is some improvement in PSNR compared to the original DCP, the correlation with the original image remains significant, leading to unstable performance. After applying the proposed method to correct the atmospheric light estimation in the DCP, the image’s overall brightness is maintained, and the contrast is significantly improved. This enhancement leads to a decrease in MSE, resulting in a higher PSNR for the dehazed image. Furthermore, a higher SSIM indicates that after applying the proposed method, the overall structure of the image more closely resembles the real scene, thus ensuring effective dehazing.

#### 5.4.2. Fattal’s with PDGA

In this subsection, we apply the proposed methodology to the conventional dehazing technique known as Fattal’s method. Fattal’s method incorporates elements such as the atmospheric transmission model, transmission function, and surface shading variable. It posits that surface shading and the transmission function are statistically uncorrelated. Building on this premise, the model computes and analyzes atmospheric scattering to estimate the transmission function and improve image clarity. However, the effectiveness of this approach is heavily dependent on the selection of atmospheric light intensity. The artificially specified atmospheric light parameters significantly influence the dehazing outcome, particularly impacting the color rendition in the dehazed image.

**Figure 14:**
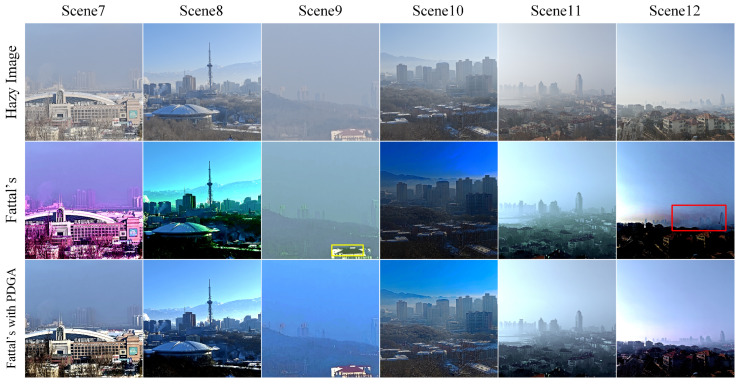
The performance of Fattal’s proposed approach.

Fattal’s approach requires the manual determination of the intensity vector of atmospheric light in the RGB color space, which signifies the distribution of the RGB channels in atmospheric light intensity. In our experimental methodology, we utilized the atmospheric light intensity vector suggested by the original authors, denoted as [0.8, 0.8, 0.9]. However, it has been observed that this recommended atmospheric light intensity vector may not be optimal for effectively dehazing natural scene images that include sky regions, leading to color distortions in the dehazed images. Figure 14 illustrates the dehazing results obtained by applying Fattal’s technique alone and by combining Fattal’s method with PDGA.

The utilization of an imprecise atmospheric light intensity vector in Fattal’s dehazing algorithm can result in significant color deviations, particularly evident in scenes 7, 8, and 9 as illustrated in Figure 14. In Scene 7, although Fattal’s dehazing algorithm shows efficacy, the heightened green component within the atmospheric light vector leads to a noticeable color shift towards purple in the resultant dehazed image. Scene 8 exhibits a predominant shift towards green in the color balance of the dehazed image due to the low green component in the atmospheric light vector. In Scene 9, the red component in the roof region, outlined by the yellow rectangular box, is notably attenuated, a consequence of the disproportionately high red component in the atmospheric light vector. Examination of the red rectangular region in Scene 12 reveals color banding when applying Fattal’s technique, a phenomenon resulting from inaccuracies in the atmospheric light vector values. This issue arises from the excessive adjustment of all three components of the atmospheric light vector, leading to a general reduction in image luminance and subsequent color banding in regions with low frequencies.

The method presented addresses the difficulties associated with Fattal’s approach, which necessitates manual specification of the atmospheric light vector. This manual input can pose challenges in effectively dehazing images with low-frequency components, like the sky. By utilizing the estimated atmospheric light intensity derived from the proposed technique as the input for Fattal’s algorithm, it becomes feasible to alleviate the problem of color distortion.

Table 2 presents the quantitative evaluation results of Fattal’s dehazing technique before and after incorporating the recommended modifications. The findings reveal that the application of Fattal’s method with PDGA yields significantly improved performance compared to Fattal’s original method across various images, except for Scene 9. Despite Scene 9 showing higher PSNR and SSIM values with Fattal’s method, it is marred by noticeable color distortions, resulting in an unrealistic display of the dehazed image. Additionally, the modified algorithm demonstrates better control over brightness, leading to increased contrast and enhanced detail in the images. The integration of PDGA is particularly noteworthy as it dynamically provides atmospheric light intensity vector parameters for Fattal’s method, enabling real-time implementation in dehazing systems. In Scene 12, Fattal’s use of a high global atmospheric light intensity vector leads to reduced brightness and contrast in the dehazed image, especially in low-energy areas of the scene. By employing the proposed method for global atmospheric light estimation, more accurate estimates are obtained, preserving the original image’s contrast while effectively dehazing the scene.

#### 5.4.3. GPLF with PDGA

In this subsection, we utilize PDGA to implement GPLF, a recent haze removal method based on the polarization properties of images. The underlying principle of GPLF is rooted in the observation that the polarized sky portion in natural scene images displays low-frequency characteristics in the frequency domain. The technique involves the application of a low-pass filter in the frequency domain to process the polarized image, with the objective of preserving low-frequency elements while eliminating high-frequency elements. Subsequent to this filtering step, the polarized image is reconstructed using the inverse Fourier transform, focusing on the sky region to determine the overall atmospheric light. Through comprehensive experimental validation, it was determined that this theoretical framework has specific constraints. In cases where there are block regions in the scene with similar polarimetric properties, these regions are retained and factored into the estimation of the global atmospheric light, potentially leading to significant estimation biases and influencing the final dehazing results.

**Figure 15:**
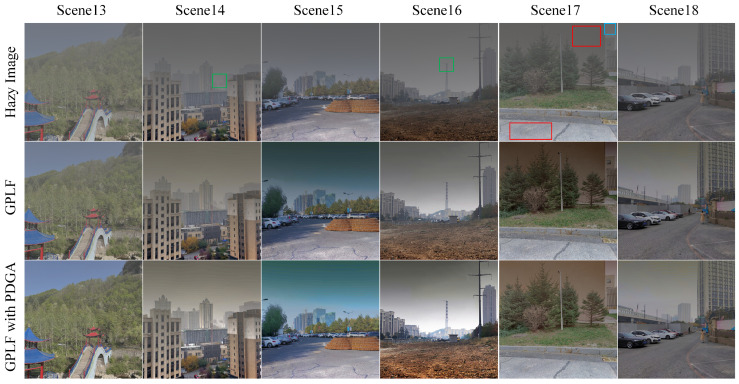
The performance of proposed approach in GPLF.

Figure 15 demonstrates the outcomes of dehazing both before and after integrating PDGA estimation to substitute the traditional global atmospheric light estimation in GPLF. The low-frequency signal characteristics in polarized images extend beyond the sky portion to specific areas of the object within the image. Consequently, certain object energies become integrated as elements of the overall atmospheric light even post low-pass filtering. Upon analysis of Scene 17 depicted in Figure 15, it is evident that the sky region is relatively limited and confined to the blue rectangular section in the upper right corner. Nevertheless, the red rectangular area also encompasses low-frequency signals. These signals are retained by the GPLF within the calculated global atmospheric light domain, potentially introducing notable estimation biases and leading to suboptimal dehazing outcomes.

**Figure 16:**
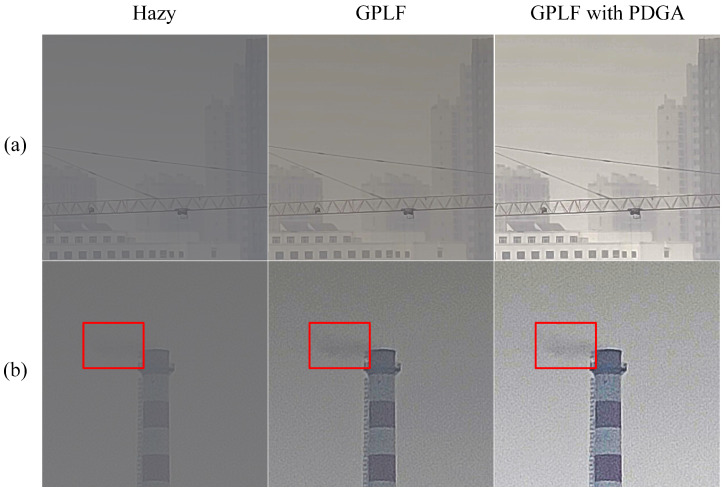
The zoomed-in views of the regions of interest marked with green rectangles in Figure 15. (**a**) Portion of Scene 14. (**b**) Portion of Scene 16.

The magnified images of the green rectangular regions in Scene 14 and Scene 16 are depicted in Figure 16. The utilization of PDGA significantly enhances the contrast of distant objects that were previously obscured by haze. The impact of this enhancement is particularly noticeable in the intensification of smoke within the red rectangular area shown in Figure 16.

Table 3 presents an impartial evaluation of the dehazing results obtained by employing GPLF technique alone and in combination with PDGA. By refining the estimation of global atmospheric light in GPLF using the proposed method, a notable enhancement in the dehazing results is observed. Notably, there is a noticeable increase in contrast compared to using GPLF alone. This indicates that after applying the proposed approach, the finer details in the image are more pronounced.

#### 5.4.4. Performance on Public Dataset

In order to illustrate the advantages of the proposed method, we incorporated various dehazing techniques alongside PDGA processing to enhance images obtained from the publicly available polarization image dataset PolarLITIS (https://zenodo.org/records/5547760, accessed on 3 March 2024). As the polarized and visible light color images in this dataset are initially free of haze, we introduced a controlled level of haze to the images using Equation (2) before applying the dehazing method. Subsequently, SSIM was utilized to objectively assess the resemblance between the dehazed images produced by each technique and the original haze-free images.

**Figure 17:**
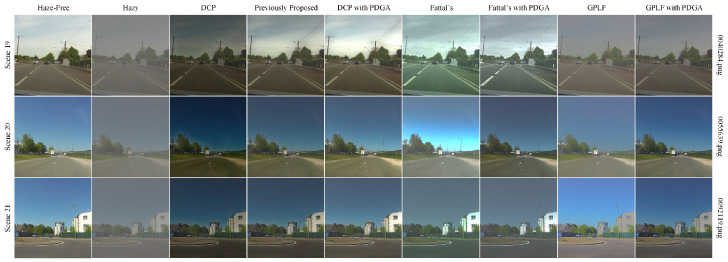
The performance of PDGA on PolarLITIS.

The impact of employing the PDGA technique for dehazing on the PolarLITIS dataset is illustrated in Figure 17. The utilization of PDGA in the three aforementioned methods leads to a notable enhancement in the dehazing effect, resulting in dehazed images closer to the original haze-free images.

Table 4 displays the similarity scores computed through the SSIM metric between the dehazed images produced by the specified algorithms and the haze-free images sourced from the PolarLITIS dataset. The results indicate that the incorporation of the proposed approach for estimating global atmospheric light leads to improvements in SSIM for all four dehazing techniques. This outcome underscores the effectiveness and adaptability of the proposed methodology.

### 5.5. Execution Time

Ultimately, an evaluation of the operational effectiveness of the proposed approach is provided. The average runtime of the algorithm is detailed in the final row of Table 1, Table 2 and Table 3.

The initial DCP method was independently applied on both CPU and GPU. As indicated in Table 1, the traditional DCP algorithm necessitates over 20 s for execution on the CPU, and our proposed methodology yields a total runtime of approximately 60 s. The increased runtime on the CPU can be attributed to the significant number of matrix operations required to calculate the polarization state for each pixel within the image. In order to achieve real-time haze removal, we parallelized the original DCP and PDGA algorithms on the GPU, resulting in a notable reduction in runtime. The processing time for DCP with PDGA on the GPU is estimated to be approximately 5 ms.

Currently, a parallelized adaptation of Fattal’s algorithm has not been integrated. Consequently, the execution time of Fattal’s algorithm as indicated in Table 2 is estimated to be around 990 ms. When employing Fattal’s algorithm in conjunction with PDGA, the algorithm is processed on the CPU while the PDGA component operates on the GPU, leading to a combined runtime of 994 ms.

The parallelization of the GPLF technique has been effectively achieved. The conventional GPLF approach requires the application of Fourier transform and inverse transform techniques to ascertain the overall atmospheric light. This procedure demonstrates a higher level of algorithmic intricacy in comparison to the calculation of polarization states in PDGA. Upon reviewing Table 3, it is evident that the runtime of GPLF combined with PDGA is shorter than that of standalone GPLF, with timings of 6.99 ms and 12.03 ms, respectively.

## 6. Conclusions

### 6.1. Achievements and Contributions

We proposed an approach for estimating atmospheric light by leveraging polarization gradient data and applied it in existing haze removal methodologies reliant on atmospheric light intensity. Our experimental results demonstrate that this method enhances the accuracy of atmospheric light intensity estimation, thereby enhancing the dehazing performance of techniques based on airlight. Based on this approach, we developed a real-time polarization dehazing system capable of executing various dehazing algorithms based on airlight, which have been optimized for parallel processing on GPU, resulting in a substantial reduction in processing time compared to the image acquisition frame rate, thereby achieving real-time dehazing capabilities.

### 6.2. Limitations of the Proposed Method

The method outlined in this study utilizes image polarization states to identify the sky area and then calculate the intensity of atmospheric light. As a result, the system’s effectiveness is variable when analyzing scenes that do not include the sky region.

**Figure 18:**
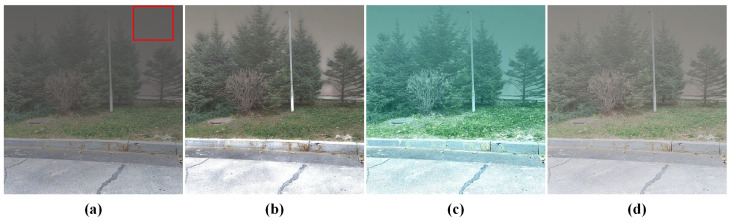
Failure case. (**a**) Foggy image without sky. (**b**) DPC with PDGA. (**c**) Fattal’s with PDGA. (**d**) GPLF with PDGA.

The impact of haze removal on the image of Scene 17 is demonstrated in Figure 18 after the sky region has been eliminated. Due to the lack of sky region in the image, the PDGA identifies the red rectangular area as the PZCI and estimates it as the atmospheric light intensity. However, this region encompasses energy emitted by objects beyond atmospheric light, resulting in the suboptimal performance of PDGA in the aforementioned algorithm.

### 6.3. Future Work

As delineated in Section 6.2, the proposed approach may not attain peak efficacy when applied to images devoid of sky regions. Consequently, we plan to introduce refinements customized for such images. These refinements will involve a quantitative examination and statistical delineation of the polarization attributes of atmospheric light constituents within scene images. This strategy is designed to differentiate atmospheric light from the scene, providing a fresh framework for estimating atmospheric light intensity. Furthermore, we will investigate techniques for utilizing polarization data derived from prevalent features such as highly polarized surfaces to estimate atmospheric light.

In addition, we have noticed that a rising trend has emerged in utilizing deep learning techniques for image dehazing, with various innovative methods demonstrating impressive dehazing capabilities. For example, Wang et al. [31] introduced the Frequency Compensation Block (FCB) to address spectral shift challenges in deep networks when learning high-frequency image patterns, thereby enhancing image detail recovery in dehazing. Nie et al. [32] unveiled the LCEFormer dehazing network, which combines LEA and LCFN techniques in a transformer architecture, featuring an adaptive local context enrichment module (ALCEM) based on CNN to enhance dehazing performance by capturing contextual information from local regions. Yuan et al. [33] proposed the Visual Transformer with Stable Prior and Patch-level Attention (VSPPA) for image dehazing, emphasizing the importance of local positional correlation in deep learning-driven dehazing processes. We plan to incorporate our methodology, with a special focus on polarization information, into deep learning-based dehazing techniques to elevate the quality of dehazing results.

## Figures and Tables

**Table 1:** Evaluations of algorithms based on DCP. Bold type to indicate the highest value and underlining to represent the second-highest value.

Scene	Evaluation Method	Hazy	DCP	Previously Proposed	DCP with PDGA
	Contrast	29.340	42.082	42.561	**53.996**
Scene 1	PSNR	-	8.666	10.827	**12.570**
	SSIM	-	0.663	0.801	**0.836**
	Contrast	0.586	3.468	6.263	**12.928**
Scene 2	PSNR	-	6.129	7.595	**10.769**
	SSIM	-	0.521	0.625	**0.736**
	Contrast	0.774	2.595	5.037	**7.726**
Scene 3	PSNR	-	7.558	9.129	**12.911**
	SSIM	-	0.581	0.682	**0.814**
	Contrast	21.146	31.964	38.644	**46.922**
Scene 4	PSNR	-	9.382	10.900	**12.293**
	SSIM	-	0.610	0.666	**0.743**
	Contrast	46.005	129.73	**377.436**	201.747
Scene 5	PSNR	-	8.044	12.2423	**13.926**
	SSIM	-	0.545	0.514	**0.671**
	Contrast	46.874	78.729	88.213	**134.082**
Scene 6	PSNR	-	10.515	13.089	**14.429**
	SSIM	-	0.689	**0.799**	0.715
Avg Time	CPU (s)	-	20.97	59.33	60.35
	GPU (ms)	-	3.99	5.02	5.04

**Table 2:** Evaluations of algorithms based on Fattal’s approach. Bold type to indicate the highest value and underlining to represent the second-highest value.

Scene	Evaluation Method	Hazy	Fattal’s	Fattal’s with PDGA
	Contrast	29.340	**134.758**	124.700
Scene 7	PSNR	-	14.303	**14.427**
	SSIM	-	0.777	**0.789**
	Contrast	25.291	70.589	**76.033**
Scene 8	PSNR	-	11.423	**13.757**
	SSIM	-	0.623	**0.741**
	Contrast	0.774	3.491	**5.176**
Scene 9	PSNR	-	**22.637**	13.990
	SSIM	-	**0.969**	0.884
	Contrast	11.085	**22.001**	21.146
Scene 10	PSNR	-	9.255	**12.451**
	SSIM	-	0.576	**0.788**
	Contrast	19.114	41.895	**42.693**
Scene 11	PSNR	-	18.343	**18.922**
	SSIM	-	0.860	**0.867**
	Contrast	**78.481**	54.816	75.406
Scene 12	PSNR	-	11.812	**15.459**
	SSIM	-	0.656	**0.782**
Avg Time (ms)		-	990@CPU	994@CPU+GPU

**Table 3:** Evaluations of algorithms based on GPLF. Bold type to indicate the highest value and underlining to represent the second-highest value.

Scene	Evaluation Method	Hazy	GPLF	GPLF with PDGA
	Contrast	74.610	143.671	**262.401**
Scene 13	PSNR	-	28.715	**30.759**
	SSIM	-	0.941	**0.953**
	Contrast	41.104	60.578	**98.622**
Scene 14	PSNR	-	29.08	**20.262**
	SSIM	-	0.981	**0.929**
	Contrast	138.257	188.508	**203.609**
Scene 15	PSNR	-	**20.872**	19.653
	SSIM	-	**0.930**	0.917
	Contrast	40.832	114.186	**315.810**
Scene 16	PSNR	-	19.532	**13.588**
	SSIM	-	0.863	**0.670**
	Contrast	226.558	302.574	**310.443**
Scene 17	PSNR	-	19.013	**25.258**
	SSIM	-	0.858	**0.905**
	Contrast	49.883	85.997	**88.976**
Scene 18	PSNR	-	27.372	**24.628**
	SSIM	-	0.944	**0.937**
Avg Time (ms)	GPU	-	12.03	6.99

**Table 4:** SSIM of algorithms on PolarLITIS.

Scene	DCP	Previously Proposed	DCP with PDGA	Fattal’s	Fattal’s with PDGA	GPLF	GPLF with PDGA
Scene 19	0.581	0.874	0.960	0.850	0.903	0.895	0.912
Scene 20	0.605	0.890	0.951	0.821	0.927	0.887	0.941
Scene 21	0.756	0.870	0.948	0.865	0.923	0.866	0.932

## Data Availability

After the publication of this article, readers are provided with the opportunity to access the data that was introduced and utilized but not explicitly presented in the article. This data can be accessed through the author’s Google Drive at the following URL: https://drive.google.com/drive/folders/13M8v_jGTNlLqebmOTJSeMFp0Fm1qTuVR. Researchers may also communicate with the corresponding author to acquire pertinent data.

## Abbreviations

The following abbreviations are used in this manuscript:

DoP	Degree of Polarization
PDGA	Atmospheric Light Estimation Using Polarization Degree Gradient
PZCI	Near-Zero Closure Interval for Polarization
PSNR	Peak Signal-to-Noise Ratio
SSIM	Structural Similarity Index
